# Name-based instructor–student interaction in medical teaching: development and psychometric evaluation of a pilot questionnaire

**DOI:** 10.3352/jeehp.2026.23.26

**Published:** 2026-08-13

**Authors:** Liam Philipp Weber, Philipp Huber, Julius Wiemschulte

**Affiliations:** 1 Medizinische Fakultät, Eberhard-Karls-Universität Tübingen, Tübingen, Germany; 2 Fachbereich 11 Medizin, Justus-Liebig-Universität Giessen, Giessen, Germany; 3 Institut für Hebammenwissenschaften, Rheinische Friedrich-Wilhelms-Universität Bonn, Bonn, Germany; Hallym University, Korea

**Keywords:** Education, medical, Factor analysis, statistical, Germany, Social interaction, Surveys and questionnaires

## Abstract

**Purpose:**

When instructors address students by name, this may be associated with students’ engagement and sense of inclusion in higher education. However, name-based instructor–student interaction has rarely been conceptualized as a multidimensional construct, and no psychometrically evaluated instrument is available for medical education. This study developed and psychometrically evaluated a pilot questionnaire assessing students’ perceived experiences of being addressed by name in medical teaching contexts.

**Methods:**

Questionnaire development followed a multistep exploratory approach that included qualitative input, iterative item refinement, and expert content review. An initial pool of 59 items was administered to medical students (n=270) from semesters 2–10 at a German medical faculty. Factor structure was examined using exploratory factor analysis based on Pearson correlations, oblique rotation, and predefined item-reduction criteria. Internal consistency was assessed using McDonald’s ω and Cronbach’s α, discriminant validity using the heterotrait–monotrait ratio, and generalized partial credit models were estimated separately for each factor.

**Results:**

Exploratory factor analysis yielded a parsimonious 3-factor solution comprising 15 items: cognitive activation, appreciation & social belonging, and social evaluative anxiety. The final model showed good fit, and all factors demonstrated strong internal consistency (ω=0.84–0.88), good measurement precision (expected a posteriori reliability=0.83–0.88), adequate discriminant validity (heterotrait–monotrait ratio <0.85), and acceptable item fit.

**Conclusion:**

The pilot questionnaire captures 3 distinct dimensions of name-based instructor–student interaction and provides promising initial psychometric evidence for medical education research. It may support confirmatory validation and further study of relational teaching practices.

## Graphical abstract

[Fig f5-jeehp-23-26]

## Introduction

### Background

Previous research suggests that students’ perceptions that instructors know them by name may be associated with important aspects of the educational experience, including engagement and a sense of inclusion [1,2]. Being known by name, however, denotes a cognitive state of recognition on the part of the instructor, whereas being addressed by name constitutes a behavioral, interactional event that students directly experience. The present study focuses on the latter, hereafter referred to as name-based instructor–student interaction. In large university courses, the use of students’ names may function as a cue to interpersonal recognition, even when sustained individualized interaction is limited [1]. In health professions education, where teaching often occurs in hierarchical and time-pressured settings, subtle interpersonal behaviors may shape learners’ willingness to participate and their interpretation of educational experiences, such as feedback and assessment [3-5]. Name-based instructor–student interaction may therefore have both facilitative and inhibitory potential: for some students, it may foster engagement and belonging, whereas for others, it may heighten self-consciousness or evaluative concern.

Despite this conceptual relevance, name-based instructor–student interaction has rarely been examined as a multidimensional construct in medical education. Prior work has often relied on isolated indicators, offering limited insight into the experiential dimensions of name-based interaction [1,2]. A psychometrically sound instrument is therefore needed to support theory-driven and evaluative research on individualized interaction in medical teaching.

### Objectives

Against this background, the present study aimed to develop and psychometrically evaluate a pilot questionnaire assessing students’ perceived experiences of being addressed by name in medical teaching contexts. Rather than testing predefined theoretical assumptions, the primary objective was to identify salient experiential domains and examine the initial measurement properties of the resulting scales. By providing an empirically grounded pilot instrument, this study seeks to support subsequent confirmatory validation and further research on relational teaching practices in health professions education.

## Methods

### Ethics statement

The study protocol was reviewed by the Ethics Committee of Justus-Liebig-Universität Giessen, which determined that formal ethical approval was not required. Participation was voluntary and anonymous, and completion of the questionnaire was regarded as indicating informed consent.

### Study design

We conducted a single-center, cross-sectional pilot questionnaire-development and psychometric-evaluation study.

### Setting

The study was conducted at the Faculty of Medicine, Justus Liebig University Giessen, Germany. Quantitative data were collected in December 2025 using a paper-based questionnaire administered after scheduled teaching sessions.

### Instrument development

Questionnaire development followed a multistep exploratory approach combining qualitative input, iterative item formulation, and expert content review; an overview of the development and analysis process is provided in Supplement 1. The study was informed by the Standards for Educational and Psychological Testing as a general psychometric framework for instrument development and evaluation [6]. Because no dedicated reporting guideline exists for studies of this type, reporting followed established recommendations for early-stage scale development and validation in health research [7]. The development team consisted of one investigator experienced in medical education research and two senior medical students.

Initial item generation drew on the development team’s teaching experience. An online survey with 4 open-ended questions was administered to 25 medical students to capture experiences of being personally addressed by instructors (Supplement 2). The responses, together with a review of relevant literature, informed the development of an initial pool of 59 items using a Likert-type response format (Supplements 3, 4). To obtain initial content-related validity evidence, the preliminary questionnaire was independently reviewed by 3 medical education researchers from different German medical faculties, who evaluated item relevance and clarity. The finalized item pool was subsequently used for quantitative data collection and psychometric evaluation.

### Participants

Students from semesters 2–10 were included (n=270; approximately 30 per semester). First-semester students, who had limited teaching experience, and final-year students, who were undergoing practical training only, were excluded. Participation was voluntary and anonymous, and no personal identifiers were collected. Standardized written instructions were provided before questionnaire completion.

### Variables

The primary variables were students’ responses to the questionnaire items assessing perceived experiences of being addressed by name by instructors in medical teaching contexts. Additional descriptive variables included age, gender, semester of study, number of courses attended in the current semester, weekly study time, the number of courses in which at least one instructor knew the student’s name, the instructor roles by which students had been addressed by name, and the teaching formats in which name-based address occurred.

### Data sources/measurement

Quantitative data were collected in December 2025 from medical students at Justus-Liebig-Universität Giessen, Germany. A quota-based sampling approach was used to achieve approximately balanced representation by semester and gender across the study sample. Data were collected using a paper-based questionnaire administered after teaching sessions. The questionnaire also assessed whether current-semester instructors knew students’ names (item 4) and the frequency and formats of prior experiences of being addressed by name (items 6 and 7). The raw data are available in Dataset 1.

### Study size

No formal power calculation was conducted; the target sample size of 270 students was chosen to allow approximately balanced quota-based recruitment across semesters 2–10 and gender and to provide an exploratory psychometric dataset for the initial 59-item pool.

### Statistical methods

All analyses were conducted in R ver. 4.3.0 (R Foundation for Statistical Computing) using the packages psych, TAM, and mirt. A random seed was set for reproducibility. The R code used for the statistical analyses is available in Dataset 2. Large language models were used to assist with R code development and debugging. Complete-case analysis was applied throughout; no participants were excluded because of missing data.

Item screening included descriptive statistics, floor effects, and corrected item–total correlations. To assess intra-individual response consistency, 1 item was intentionally duplicated (items X32 and X41); the second occurrence was excluded before further analyses. Data suitability for factor analysis was evaluated using the Kaiser-Meyer-Olkin (KMO) measure and Bartlett’s test of sphericity.

The latent structure was examined using exploratory factor analysis based on Pearson correlations, principal axis factoring, and oblimin rotation [8]. Pearson correlations were considered appropriate given the 5-point response format and only moderate item skewness (maximum |skewness| <1) [8]; they also provide more stable estimates than polychoric correlations for the 1,326 item pairs in the 52-item pool at the present sample size. The number of factors was determined by parallel analysis with 100 iterations, supplemented by scree plot inspection and interpretability; factors represented by fewer than 3 items after item reduction were discarded. Items were retained according to predefined criteria: primary loading ≥0.50, cross-loading ≤0.30, loading difference ≥0.20, and communality ≥0.30, with a maximum of 5 items per factor to maintain scale parsimony and limit respondent burden. Model fit was evaluated using the root mean square residual (RMSR), Tucker-Lewis index (TLI), and root mean square error of approximation (RMSEA). The 5-item solution per factor was additionally examined by comparing 5- versus 4-item generalized partial credit models and confirming that each retained item contributed positively to expected a posteriori reliability and peak test information.

Internal consistency was assessed using McDonald’s ω and Cronbach’s α. Discriminant validity was evaluated using the heterotrait–monotrait ratio, with a threshold of <0.85.

Model selection between the partial credit model and the generalized partial credit model was based on factor-specific likelihood-ratio tests. Accordingly, generalized partial credit models were estimated separately for each factor. Person–item targeting was visualized using Wright maps. Local independence was evaluated using the adjusted Yen’s Q3 statistic (Q3*=Q3−mean Q3), comparing observed maxima with a cutoff of 0.20 and with empirical 95% critical values from a parametric bootstrap with 500 replications [9].

## Results

### Participant characteristics

Participant characteristics are presented in Table 1.

### Item screening and factorability

Regarding exposure to name-based interaction, 144 participants (53.3%) reported that no instructor knew their name in a current-semester course, 95 (35.2%) reported one such course, and 31 (11.5%) reported 2 or more. Across items 6 and 7, 265 participants (98.1%) reported having been addressed by name by at least 1 instructor role or in at least 1 teaching format. Analyses were initially conducted on the full 59-item pool. The intentionally duplicated item showed acceptable response stability (r=0.72) and was excluded. Item-level screening identified 6 items with very limited variance (standard deviation ≤0.70), pronounced floor effects (>50% of responses in the lowest category), and very low corrected item–total correlations (r<0.10); these items were removed before factor analysis, leaving 52 items for subsequent analyses (Dataset 1).

The remaining item set demonstrated excellent sampling adequacy (KMO=0.91; all item-level KMO values were above accepted thresholds) and a highly significant Bartlett’s test of sphericity (χ^2^(1,326)=8,193.55, P<0.001), supporting its suitability for exploratory factor analysis.

### Factor structure and item reduction

Parallel analysis suggested 5 factors (Fig. 1). An initial exploratory factor analysis with 5 factors showed acceptable overall fit (RMSR=0.04, TLI=0.84, RMSEA=0.054). The fifth factor, tentatively reflecting peer interaction within the learning group, was represented by only 2 items in the full item pool. Factor 4, capturing students’ perceptions of their instructor, yielded only 2 items with primary loadings ≥0.50 after item reduction. Both factors were therefore discarded, yielding a 3-factor working model.

The item set was subsequently reduced according to predefined criteria: primary loading ≥0.50, cross-loading ≤0.30, loading difference ≥0.20, and communality ≥0.30, with a maximum of 5 items per factor. Factors represented by fewer than 3 items after reduction were discarded. The final model was re-estimated using the 15 retained items and demonstrated good overall fit (RMSR=0.027, TLI=0.966, RMSEA=0.047), as well as a clear factor structure with strong primary loadings and minimal cross-loadings (Fig. 2). Factor correlations were moderate between F1 and F2 (r=0.57) and negligible between F3 and F1/F2 (r=0.09 and −0.10, respectively), supporting oblique rotation and indicating related yet largely independent dimensions.

The 3 factors were labeled cognitive activation (F1), appreciation & social belonging (F2), and social evaluative anxiety (F3). Factor loadings and item assignments are displayed in Fig. 2, and full item wordings are provided in Supplement 5. A sensitivity analysis restricted to participants whose instructors knew their names in current-semester courses (n=126) replicated the 3-factor structure: all items loaded highest on their assigned factor, 13 of 15 primary loadings were ≥0.50, internal consistency was satisfactory (α=0.83–0.85), and scale means closely matched those of the remaining sample (differences ≤0.08).

### Reliability and discriminant validity

All 3 factors demonstrated good internal consistency and discriminant validity (Table 2). McDonald’s ω ranged from 0.84 to 0.88, and Cronbach’s α ranged from 0.84 to 0.88. All heterotrait–monotrait ratio values were below the conservative threshold of 0.85, supporting discriminant validity for all factor pairs. Average variance extracted exceeded 0.50 for all factors, indicating that more than half of the item variance was accounted for by the respective latent factor.

### Item response theory model fit and reliability

Factor-specific likelihood-ratio tests consistently favored the generalized partial credit model (GPCM) over the partial credit model: F1: χ^2^(5)=113.29; F2: χ^2^(5)=75.97; F3: χ^2^(5)=24.38 (all P<0.001). GPCMs were therefore used for all subsequent item response theory analyses.

Expected a posteriori reliabilities indicated good measurement precision for all 3 factors (F1: 0.880; F2: 0.831; F3: 0.840). Item fit assessed using S-X2 statistics was acceptable for most items across all factors. Test information functions showed the highest measurement precision at moderate trait levels, with peak information values of 12.02 for F1, 12.93 for F2, and 7.29 for F3 (Fig. 3). Person–item targeting and threshold ordering are displayed in the Wright maps (Fig. 4). The overlap between person locations and item threshold locations indicates that the items broadly covered the observed range of students’ perceptions.

The retention of 5 items per factor was further supported by sensitivity analyses showing that removal of the worst-fitting item consistently reduced both expected a posteriori reliability and peak test information across all factors. Maximum adjusted Q3 values were 0.199 for F1, 0.182 for F2, and 0.173 for F3; all were below 0.20 and below the empirical 95% critical values (0.213, 0.234, and 0.185), indicating no local dependence [9].

## Discussion

### Key results

From an initial pool of 59 items, exploratory factor analysis yielded a parsimonious 3-factor solution comprising 15 items. The 3 dimensions—cognitive activation, appreciation & social belonging, and social evaluative anxiety—showed good internal consistency (ω=0.84–0.88), measurement precision (EAP reliability=0.83–0.88), and discriminant validity (HTMT <0.85), with no evidence of local dependence. Taken together, these findings provide promising initial psychometric evidence for a three-dimensional representation of name-based instructor–student interaction.

### Interpretation of the factor structure

The 3-factor solution captures conceptually distinct yet complementary dimensions of how name-based instructor–student interaction may shape students’ learning experiences.

Factor 1, cognitive activation, suggests that addressing students by name may be associated with sustained attention and cognitive engagement with learning content. Personal address is a form of verbal immediacy, which has been consistently linked to student motivation [10] and to teacher support and engagement in higher education [11].

Factor 2, appreciation & social belonging, may reflect students’ sense of being individually visible and socially valued within the learning group. This interpretation aligns with conceptualizations of belonging in higher education that identify being valued and accepted as core dimensions of belonging [12], as well as with evidence showing predominantly positive, though heterogeneous, associations between sense of belonging and academic outcomes in postsecondary education [13].

Factor 3, social evaluative anxiety, appears to capture concerns about negative judgment and heightened self-consciousness when students are personally addressed in publicly visible situations. Recent evidence indicates that publicly evaluative classroom practices are associated with elevated anxiety, with fear of negative evaluation emerging as a key predictor [14]. This interpretation aligns with the literature on psychological safety [15]: depending on context and tone, name-based address may function either as a signal of recognition or as a cue of evaluative exposure. Taken together, these findings suggest that addressing students by name may simultaneously foster cognitive engagement and perceived recognition while also activating evaluative concerns, indicating a differentiated rather than uniform effect.

### Strengths

This study has several methodological strengths. Development combined qualitative input, iterative item formulation, and expert review [7], while psychometric evaluation integrated classical test theory and item response theory. Model selection, comparison with shorter alternatives, and local-independence testing all followed predefined criteria.

### Limitations

Several limitations should also be acknowledged. First, data were collected at a single medical faculty, which limits generalizability beyond the local institutional context. Second, the study was explicitly exploratory; accordingly, the identified 3-factor structure should be regarded as preliminary until it has been tested in independent samples. Third, all comparative judgments relied on retrospective self-report across heterogeneous and variably distant teaching situations. Although 98.1% of participants reported prior experience of being addressed by name, the intensity and recency of this exposure varied, and the sensitivity analysis in the subgroup whose instructors knew their names in current-semester courses should be complemented by prospective validation under documented exposure conditions. Fourth, although the development process incorporated qualitative input and expert review, further validity evidence remains necessary, particularly regarding response processes and relationships with external variables. Fifth, 2 additional content domains—students’ perceptions of their instructor and peer interaction within the learning group—could not be retained because they were insufficiently represented in the initial item pool.

Finally, because factor identification, item reduction, and evaluation of the reduced instrument were performed in the same sample, the reported psychometric indices may be optimistically biased.

### Suggestion for further studies

The content domains may warrant targeted item development in future instrument refinement.

A planned multicenter study across German medical faculties will test the factor structure using confirmatory factor analysis and evaluate measurement invariance across institutions, curriculum stages, and gender. This work could also provide reference values across teaching contexts.

### Implications for medical education

Name-based interaction may represent a previously underexamined dimension of relational teaching. Where structural anonymity is common, deliberate use of students’ names may function as a low-barrier practice associated with engagement and social recognition.

The relative independence of social evaluative anxiety from the other 2 dimensions suggests that personally addressing students may support engagement and recognition without necessarily reducing evaluative concern. This highlights the importance of considering not only whether names are used, but also how, when, and with what instructional tone they are used.

These findings are consistent with relational perspectives in health professions education, which emphasize that student–teacher interactions shape learners’ experiences of participation, feedback, and assessment [4,5]. The present instrument may therefore provide a useful framework for examining name-based address as 1 specific component of relational teaching practice. It may help future research distinguish between potentially supportive and potentially burdensome forms of individualized instructor behavior across different teaching contexts.

### Conclusion

Given its exploratory psychometric focus, this study represents a first step in measuring students’ perceptions of name-based address. Future studies should examine how contextual conditions—such as group size, familiarity, voluntariness, assessment climate, and instructional tone—shape whether name-based interaction is experienced as recognition, cognitive activation, or evaluative exposure. Accordingly, recommendations for practice should remain cautious until context-sensitive evidence is available.

## Figures and Tables

**Fig. 1.**
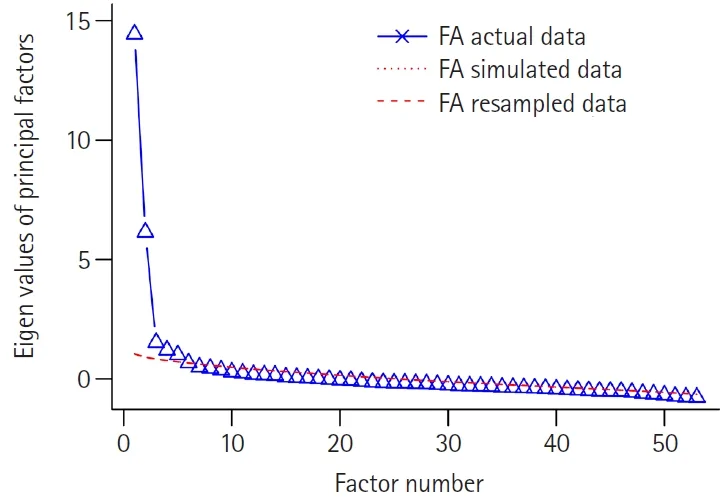
Parallel analysis scree plot for determining the number of factors. The plot shows eigenvalues from the observed data in comparison with eigenvalues obtained from simulated and resampled datasets. Factors were retained when observed eigenvalues exceeded the corresponding simulated and resampled eigenvalues.

**Fig. 2.**
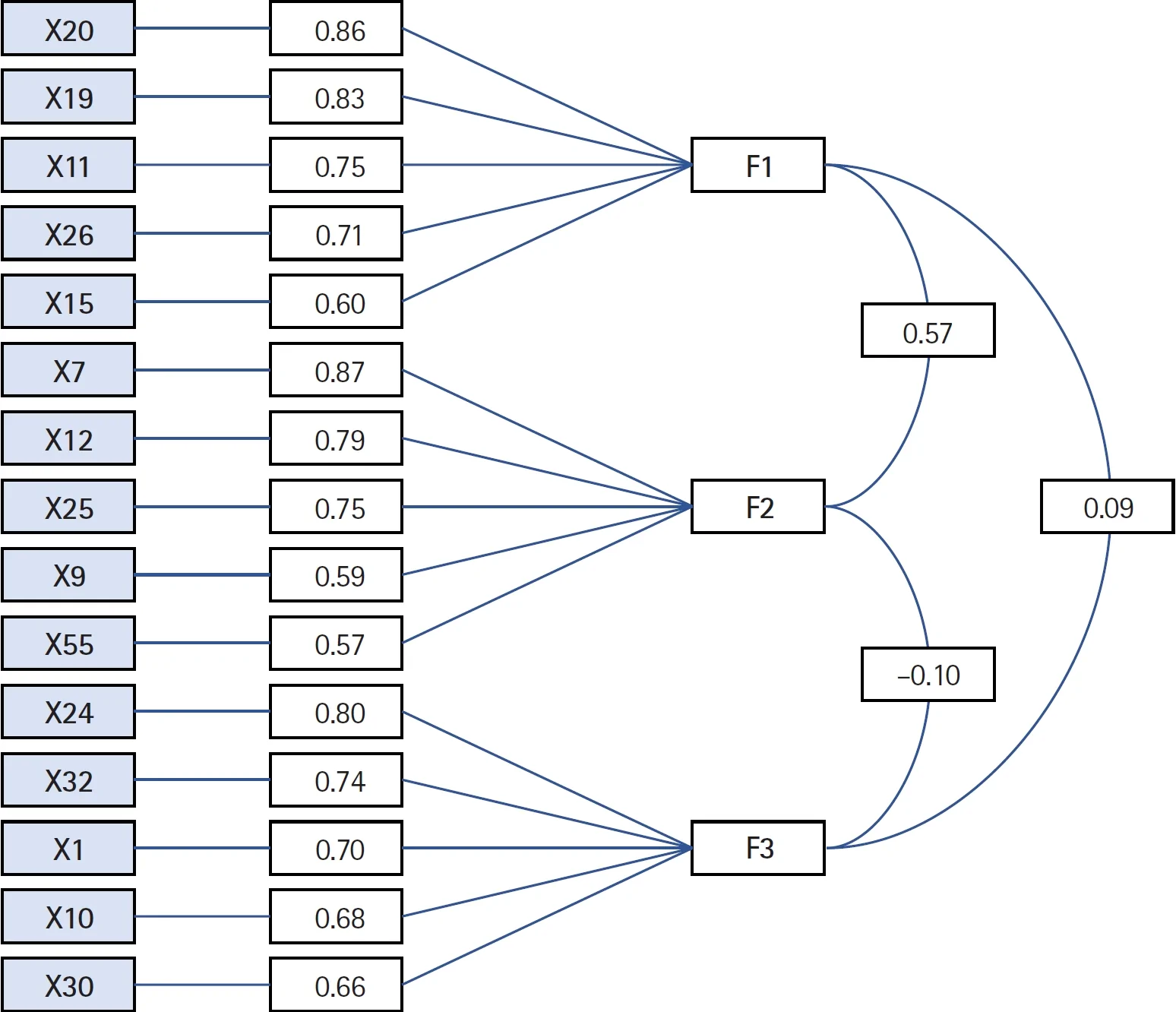
Exploratory factor analysis path diagram of the final 3-factor solution. The diagram displays the primary standardized factor loadings of the 15 retained items on Factors 1–3, as well as correlations between the latent factors. Only primary loadings are shown.

**Fig. 3.**
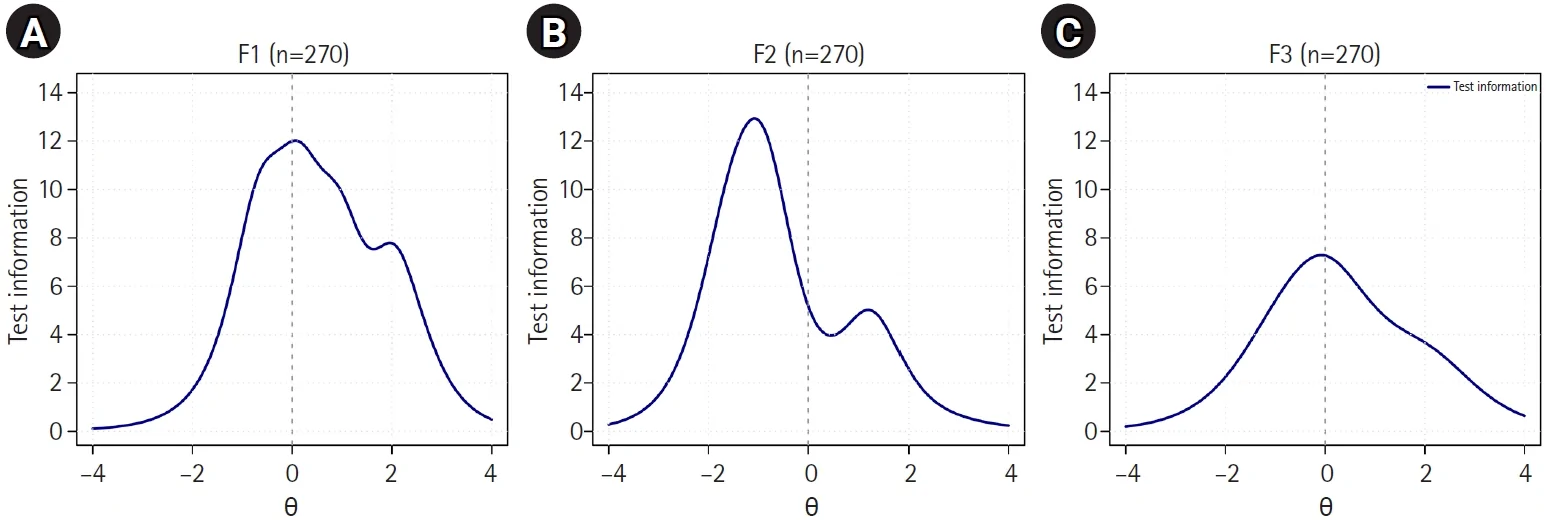
(A–C) Test information functions for Factors 1–3 of the final 15-item instrument based on generalized partial credit models (GPCMs). For each factor, the curve depicts test information across the latent trait continuum (θ). Higher values indicate greater measurement precision.

**Fig. 4.**
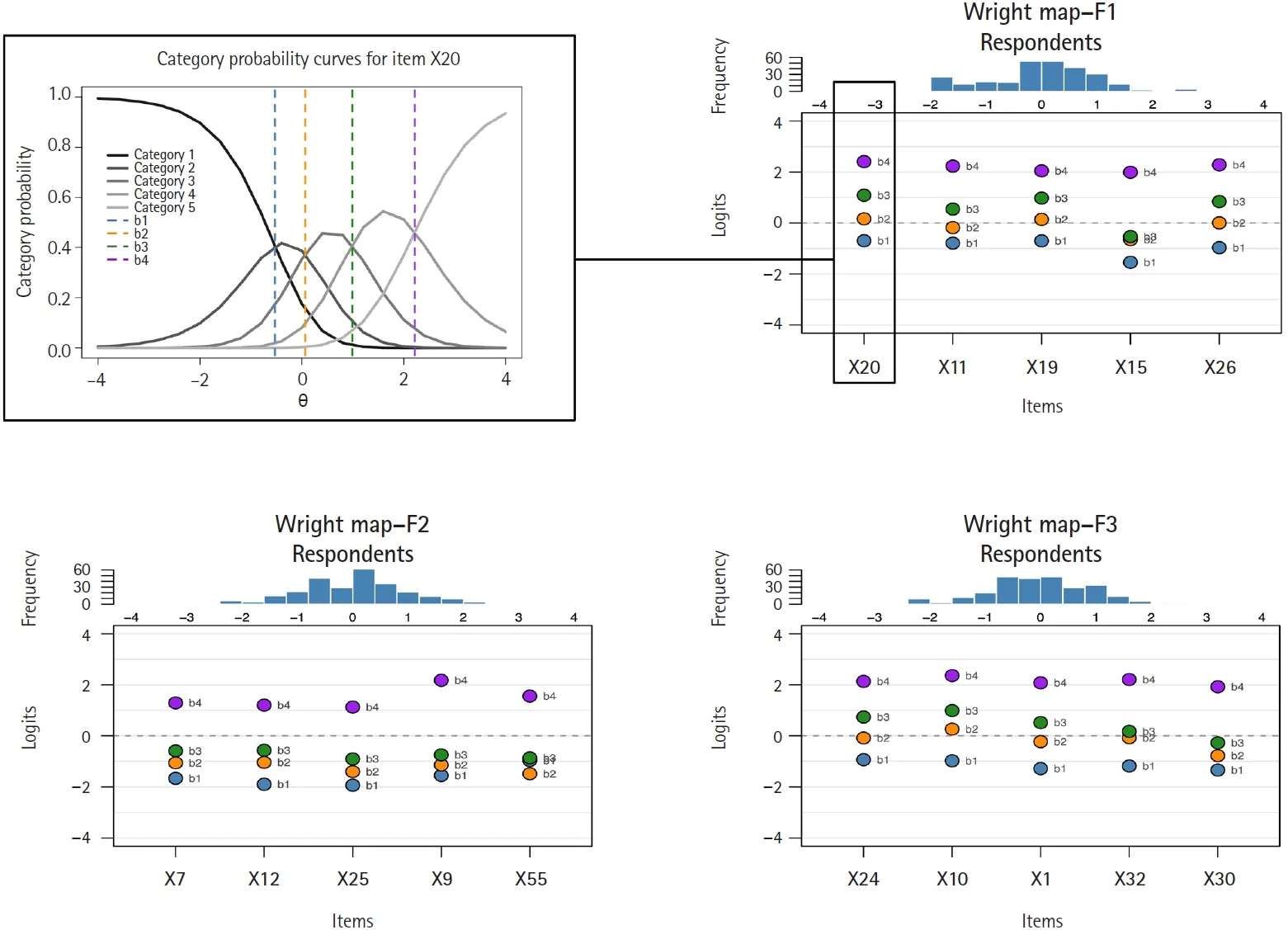
Wright maps, person–item maps, and category probability curves derived from generalized partial credit models for Factors 1–3 of the final 15-item instrument. For each factor, the Wright map displays the distribution of person locations and item threshold locations across the latent trait continuum.

**Figure f5-jeehp-23-26:**
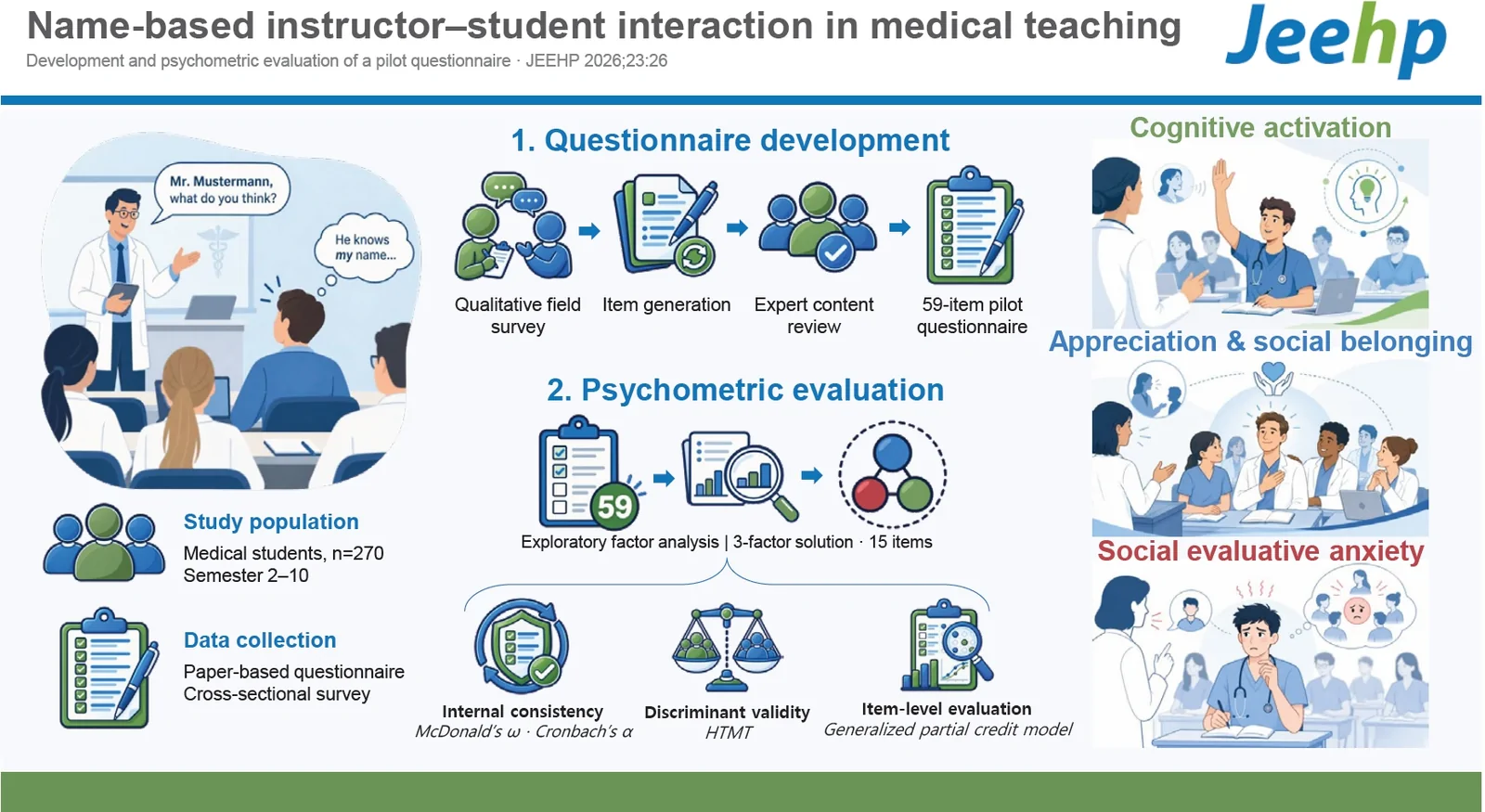


**Table 1.** Characteristics of the study participants (n=270)

Characteristic	Value
Age (yr)	23.8 (3.7) (19–50)
Gender	
Male	135 (50)
Female	135 (50)
Courses attended	6.7 (2.9)
Weekly study time (hr/wk)	3.2 (1.2)

Values are presented as mean (standard deviation) with ranges or number (%).

**Table 2.** Reliability estimates and discriminant validity indices for the 3-factor solution

Factor	Items	α	ω	AVE	HTMT F1	HTMT F2
F1: Cognitive activation	5	0.881	0.884	0.616	-	-
F2: Appreciation & social belonging	5	0.852	0.859	0.563	0.652	-
F3: Social evaluative anxiety	5	0.841	0.842	0.527	0.135	0.120

AVE, average variance extracted; HTMT, heterotrait–monotrait ratio. HTMT <0.85 indicates discriminant validity.

## Footnotes

**Data availability**
Data files are available from Harvard Dataverse: https://doi.org/10.7910/DVN/SZQHAO Dataset 1. Anonymized raw responses from the pilot questionnaire study (n=270).

Dataset 2. R code for psychometric analysis of the pilot questionnaire data.

## Supplementary materials

Supplementary files are available from Harvard Dataverse: https://doi.org/10.7910/DVN/SZQHAO

Supplement 1. Flowchart.

Supplement 2. Open questions (German and English).

Supplement 3. Pilotfragebogen Namenskenntnis (German).

Supplement 4. Pilot questionnaire (English).

Supplement 5. Item wording table.

Supplement 6. Audio recording of the abstract.
