# Supplementary material for: Name-based instructor–student interaction in medical teaching: development and psychometric evaluation of a pilot questionnaire
Source: J Educ Eval Health Prof. 2026 Aug 13;23:26. doi: 10.3352/jeehp.2026.23.26 (PMC13690480; doi:10.3352/jeehp.2026.23.26)
Supplement: Supplementary file 1 [file jeehp-23-26-suppl1.pdf]

Initial item pool (n = 59 items)

Generated from qualitative input + expert content review
Administered to N = 270 medical students

Pre-EFA item screening (59 → 52 items)

Very low variance ($SD \approx \leq 0.70$)
Pronounced floor effects (>50–75%)
Corrected item–total correlation $r < .10$
Excluded: X2, X4, X6, X17, X22, X27
To assess intra-individual response consistency, one item was intentionally duplicated (items X32 and X41) and later removed.

Factorability check (52 items)

KMO = 0.91
Bartlett's $\chi^2(1326) = 8193.55$, $p < .001$

Initial EFA (52 items)

Principal axis factoring, oblique rotation
Parallel analysis suggested 5 factors
Decision: 3-factor model

ative EFA-based item reduction

Primary loading ≥ 0.50
Cross-loading ≤ 0.30
Δ loading ≥ 0.20
Communality $h^2 \geq 0.30$

Final EFA solution (n = 15 items; 3 factors)

F1–F3: 5 items each
RMSR ≈ 0.03 | TLI ≈ 0.97 | RMSEA ≈ 0.047

Reliability and Rasch GPCM confirmation (15 items)

McDonald's ω: F1–3 = 0.84–0.88
GPCM EAP reliability: F1–3 = 0.83–0.88

IRT diagnostic evaluation

Yen's Q3: acceptable local independence
Test Information Functions: adequate measurement precision across latent trait levels
Wright maps: satisfactory person–item targeting
