# Supplementary material for: Name-based instructor–student interaction in medical teaching: development and psychometric evaluation of a pilot questionnaire
Source: J Educ Eval Health Prof. 2026 Aug 13;23:26. doi: 10.3352/jeehp.2026.23.26 (PMC13690480; doi:10.3352/jeehp.2026.23.26)
Supplement: Supplementary file 2 [file jeehp-23-26-suppl2.pdf]

Fragebogen Namensstudie zur Itemgenerierung

In welchem Fachsemester sind Sie?

Was studieren Sie?

An welcher Universität studieren Sie?

Welche Auswirkungen hat es, wenn Lehrende die Namen der Studierenden kennen?

Welche Auswirkungen hat es, wenn Studierende in Lehrveranstaltungen mit Namen angesprochen werden?

Wie wirkt sich das auf Einstellungen und Verhalten in Lehrsituationen aus?

Gibt es weitere Aspekte, die in diesem Zusammenhang wichtig sind?

Questionnaire on the 'Names Study' for item generation

Which semester are you in?

What are you studying?

Which university are you studying at?

What are the implications of lecturers knowing students' names?

What are the implications of students being addressed by name in lectures?

How does this affect attitudes and behaviour in teaching situations?

Are there any other aspects that are important in this context?
