# Supplementary material for: Name-based instructor–student interaction in medical teaching: development and psychometric evaluation of a pilot questionnaire
Source: J Educ Eval Health Prof. 2026 Aug 13;23:26. doi: 10.3352/jeehp.2026.23.26 (PMC13690480; doi:10.3352/jeehp.2026.23.26)
Supplement: Supplementary file 3 [file jeehp-23-26-suppl3.pdf]

JUSTUS-LIEBIG-
UNIVERSITÄT
GIESSEN

Fragebogen zur Namenskenntnis von Dozierenden

Hinweise zur Studie: Wir untersuchen, wie sich die Nennung des Namens durch Dozierende auf Ihr Lernklima, Ihre Motivation und Ihre Leistung auswirken kann Die Bearbeitungsdauer des Fragebogens beträgt ca. 10–15 Minuten. Bitte nehmen Sie sich ausreichend Zeit und beantworten Sie jede Frage nach bestem Gewissen.

- Die erhobenen Daten werden anonymisiert erfasst.
- Eine Rückverfolgung auf einzelne Personen ist nach Abschluss der Studie nicht möglich.

Ihre Teilnahme ist freiwillig. Sie können jederzeit ohne Angaben von Gründen abbrechen.
Zur besseren Lesbarkeit wird in diesem Fragebogen das generische Maskulinum verwendet; sämtliche Personenbezeichnungen schließen ausdrücklich alle Geschlechter mit ein.

1) **Wie alt sind Sie?**

____ Jahre

2) **Welchem Geschlecht fühlen Sie sich zugehörig?**

☐ Weiblich ☐ Männlich ☐ Divers ☐ Keine Angabe

3) **In diesem Semester besuche ich _____ Kurse. (Anzahl der Kurse)**

4) **In wie vielen dieser Kurse kennt mindestens 1 Dozent ihren Namen?**

☐ 0 ☐ 1 ☐ 2 ☐ 3 ☐ > 4

5) **Wie viel Zeit investieren Sie insgesamt pro Woche in Ihr Studium (Vorlesungen, Praktika, Selbststudium)?**

☐ Weniger als 10h ☐ 10–20h ☐ 20–30h ☐ 30–40h ☐ Mehr als 40h

6) **Bitte geben Sie für jede Rolle an, wie häufig diese Sie namentlich angesprochen hat**

	Nie	Selten	Gelegentlich	Oft	Sehr oft
Professor/Professorin	☐	☐	☐	☐	☐
Oberarzt/Oberärztin	☐	☐	☐	☐	☐
Assistenarzt/-ärztin	☐	☐	☐	☐	☐
Hochschuldozierende	☐	☐	☐	☐	☐
Student. Hilfskraft	☐	☐	☐	☐	☐

7) **Bitte wählen Sie aus, in welchen Formaten Sie namentlich angesprochen wurden (Mehrfachauswahl):**

☐ Vorlesungen (große Gruppen) ☐ Seminare/Übungen/Gruppenarbeiten
☐ Praktika/Blockveranstaltungen ☐ Klinischer Unterricht/Stationsarbeit
☐ Online-Termine/Videokonferenzen ☐ Eins-zu-Eins-Gespräche mit Dozierenden
☐ Sonstiges: _______________

8) **In welchem Format passierte Namensnennung am häufigsten**?
______________ [Auswahl aus obiger Liste]

JUSTUS-LIEBIG-UNIVERSITÄT GIESSEN

Wahrgenommene Auswirkungen

WICHTIG: Die folgenden Fragen beziehen sich darauf, ob Ihr Name in einer Veranstaltung genannt wurde oder nicht.

Bitte rufen Sie sich zur Beantwortung **mehrere typische Situationen** ins Gedächtnis – idealerweise mindestens:

- **drei Veranstaltungen mit Namensnennung**, und
- **drei Veranstaltungen ohne Namensnennung**.

Nutzen Sie dabei nach Möglichkeit verschiedene Lehrformate (z. B. Vorlesungen, Seminare, Praktika/Blockveranstaltungen, klinischer Unterricht, Online-Termine).
Alle folgenden Aussagen beziehen sich darauf, wie Sie sich in Veranstaltungen MIT Namensnennung im Vergleich zu Veranstaltungen OHNE Namensnennung fühlen bzw. verhalten.

	Stimme überhaupt nicht zu	Stimme nicht zu	Teils/Teils	Stimme zu	Stimme voll und ganz zu
Ich habe Angst, dass Falschaussagen mit meiner Person in Verbindung gebracht werden.	☐	☐	☐	☐	☐
Namensnennung finde ich aus kulturellen Gründen unangenehm.	☐	☐	☐	☐	☐
In der Veranstaltung fühle ich mich wohler.	☐	☐	☐	☐	☐
Ich finde es unangenehm, wenn mein Name in der Seminargruppe genannt wird.	☐	☐	☐	☐	☐
Ich fühle mich besser auf Prüfungen vorbereitet.	☐	☐	☐	☐	☐
Namensnennung finde ich aus persönlichen Gründen unangenehm.	☐	☐	☐	☐	☐
Ich fühle mich wertgeschätzter.	☐	☐	☐	☐	☐
Ich muss mich mehr überwinden, einen Beitrag zu leisten.	☐	☐	☐	☐	☐
Ich fühle mich der Lerngruppe/dem Kurs zugehöriger.	☐	☐	☐	☐	☐
Wenn ich etwas Falsches sage, denken meine Mitstudierenden schlecht über mich.	☐	☐	☐	☐	☐
Ich kann mir die Inhalte längerfristiger merken.	☐	☐	☐	☐	☐

	Stimme überhaupt nicht zu	Stimme nicht zu	Teils/Teils	Stimme zu	Stimme voll und ganz zu
Ich fühle mich als Person ernstgenommener.	☐	☐	☐	☐	☐
Ich finde Namensnennung unangenehm, weil ich mich in sozialen Situationen leicht unsicher fühle.	☐	☐	☐	☐	☐
Die Lernumgebung fühlt sich sicherer an.	☐	☐	☐	☐	☐
Ich bin konzentrierter.	☐	☐	☐	☐	☐
Ich habe das Gefühl, dass meine Beiträge ernster genommen werden.	☐	☐	☐	☐	☐
Durch die Namensnennung wird in meine Privatsphäre eingegriffen.	☐	☐	☐	☐	☐
Ich fühle mich unter Druck gesetzt, mich zu äußern.	☐	☐	☐	☐	☐
Ich interessiere mich dadurch mehr für die Inhalte des Fachs.	☐	☐	☐	☐	☐
Ich verstehe die Inhalte besser.	☐	☐	☐	☐	☐
Ich möchte den Dozenten nicht mit meiner Leistung enttäuschen.	☐	☐	☐	☐	☐
Namensnennung empfinde ich als aufdringlich.	☐	☐	☐	☐	☐
Es ist einfacher, Fehler zu machen und Fragen zu stellen.	☐	☐	☐	☐	☐
Wenn ich etwas Falsches sage, denkt der Dozent schlecht über mich.	☐	☐	☐	☐	☐
Ich fühle mich als individuelle Person wahrgenommen.	☐	☐	☐	☐	☐
Meine Sicherheit für folgende Prüfungen nimmt zu.	☐	☐	☐	☐	☐
Ich bleibe lieber anonym, sodass ich der Veranstaltung stumm folgen kann.	☐	☐	☐	☐	☐

JUSTUS-LIEBIG-UNIVERSITÄT GIESSEN

	Stimme überhaupt nicht zu	Stimme nicht zu	Teils/Teils	Stimme zu	Stimme voll und ganz zu
Ich komme durch Namensnennung öfter mit Kommilitonen außerhalb der Veranstaltung ins Gespräch.	☐	☐	☐	☐	☐
Ich kann meinen Lernfortschritt mehr reflektieren.	☐	☐	☐	☐	☐
Ich werde nervös, dass ich eher aufgerufen werden könnte.	☐	☐	☐	☐	☐
Ich werde mir meiner eigenen Präsenz im Raum stärker bewusst.	☐	☐	☐	☐	☐
Ich habe das Gefühl, von Kommilitonen stärker bewertet zu werden.	☐	☐	☐	☐	☐
Ich traue mich lieber, Fragen zu stellen.	☐	☐	☐	☐	☐
Ich versuche soziale Normen sorgsamer zu erfüllen (z. B. Pünktlichkeit).	☐	☐	☐	☐	☐
Ich beteilige mich häufiger.	☐	☐	☐	☐	☐
Ich höre aufmerksamer zu.	☐	☐	☐	☐	☐
Ich investiere mehr Zeit in die Vor- und Nachbereitung.	☐	☐	☐	☐	☐
Ich komme durch Namensnennung öfter mit Kommilitonen während der Veranstaltung ins Gespräch.	☐	☐	☐	☐	☐
Ich nutze Fehltermine weniger.	☐	☐	☐	☐	☐
Ich unterlasse ablenkende Tätigkeiten (z. B. Handynutzung).	☐	☐	☐	☐	☐
Ich habe das Gefühl, von Kommilitonen stärker bewertet zu werden.	☐	☐	☐	☐	☐
Die Hemmschwelle, dem Dozenten persönliche Probleme entgegenzubringen, sinkt dadurch.	☐	☐	☐	☐	☐
Die Veranstaltung wird dadurch interaktiver.	☐	☐	☐	☐	☐

	Stimme überhaupt nicht zu	Stimme nicht zu	Teils/Teils	Stimme zu	Stimme voll und ganz zu
Der Dozent spricht mich eher an, wenn er glaubt, dass ich Wissensdefizite habe.	☐	☐	☐	☐	☐
Ich nehme Sprechstundenangebote eher wahr.	☐	☐	☐	☐	☐
Namensnennung weckt in mir die Hoffnung, dass sich Vorteile im Studienverlauf ergeben (z. B. Promotion, Praktikum).	☐	☐	☐	☐	☐
Meine Vorkenntnisse werden eher berücksichtigt.	☐	☐	☐	☐	☐
Die Veranstaltung wird dadurch strukturierter.	☐	☐	☐	☐	☐
Meine Lernerfolge werden eher gelobt.	☐	☐	☐	☐	☐
Der Dozent spricht mich gezielter an, um mein Wissen zu überprüfen.	☐	☐	☐	☐	☐
Ich habe das Gefühl, dass namentlich bekannte Studierende von Dozierenden bevorzugt werden.	☐	☐	☐	☐	☐
Der Dozent erscheint kompetenter.	☐	☐	☐	☐	☐
Die persönliche Ansprache steigert meine Motivation, mich mit den Inhalten zu beschäftigen.	☐	☐	☐	☐	☐
Ich suche direkt davor oder danach eher das individuelle Gespräch mit dem Dozierenden.	☐	☐	☐	☐	☐
Der Dozent wird dadurch sympathischer.	☐	☐	☐	☐	☐
Ich habe das Gefühl, dass mein Lernerfolg für den Dozenten wichtiger ist.	☐	☐	☐	☐	☐
Die Ansprache erfolgt selektiv, sodass mein individueller Wissenstand berücksichtigt wird.	☐	☐	☐	☐	☐
Ich werde häufiger angesprochen.	☐	☐	☐	☐	☐
Ich habe das Gefühl, der Dozent möchte mich herauspicken.	☐	☐	☐	☐	☐
