# Supplementary material for: Name-based instructor–student interaction in medical teaching: development and psychometric evaluation of a pilot questionnaire
Source: J Educ Eval Health Prof. 2026 Aug 13;23:26. doi: 10.3352/jeehp.2026.23.26 (PMC13690480; doi:10.3352/jeehp.2026.23.26)
Supplement: Supplementary file 4 [file jeehp-23-26-suppl4.pdf]

JUSTUS-LIEBIG-UNIVERSITÄT GIESSEN

Questionaire on Instructors‘ Knowledge of Students’ Names

Study information: We are investigating how being addressed by name by instructors may influence your learning climate, your motivation, and your performance. The completion time of the questionnaire is approximately 10–15 minutes. Please take sufficient time and answer each question to the best of your knowledge.

• The collected data will be recorded anonymously.

• It will not be possible to trace responses back to individual persons after completion of the study.

Participation is voluntary. You may withdraw at any time without giving reasons.

For reasons of readability, the generic masculine form is used in this questionnaire; all personal designations explicitly include all genders.

1) How old are you?

_____ years

2) Which gender do you identify with?

☐ Female ☐ Male ☐ Diverse ☐ Prefer not to say

3) This semester, I am attending _____ courses. (Number of courses)

4) In how many of these courses does at least one instructor know your name?

☐ 0 ☐ 1 ☐ 2 ☐ 3 ☐ > 4

5) How much total time do you invest in your studies per week (lectures, practical courses, self-study)?

☐ Less than 10h ☐ 10–20h ☐ 20–30h ☐ 30–40h ☐ More than 40h

6) Please indicate how often each role has addressed you by name

	Never	Rarely	Sometimes	Often	Very often
Professor	☐	☐	☐	☐	☐
Senior Physician	☐	☐	☐	☐	☐
Resident Physician	☐	☐	☐	☐	☐
University lecturer	☐	☐	☐	☐	☐
Student assistant	☐	☐	☐	☐	☐

7) Please select the formats in which you habe been addressed by name (multiple selection):

☐ Lectures (large groups) ☐ Seminares/Exercises/Group work
☐ Practical courses/block courses ☐ Clinical teaching/ward-based teaching
☐ Online sessions/Video conferences ☐ One-to-One conversations
☐ Other: _______________

8) **In which format did name-based address occur most frequently?**

______________ [Choose from the list above]

Perceived Effects

Important: The following questions refer to whether your name was mentioned in a session or not.
To answer the question, please recall several typical situations – ideally at least:

- **three sessions with name mention,** and
- **three sessions without name mention.**

Whenever possible, use different teaching formats (e.g., lectures, seminars, practical/block courses, clinical teaching, online sessions).

All oft he following statements refer to how you feel or behave in sessions WITH name mention compared sessions WITHOUT name mention.

	Strongly disagree	Disagree	Neither agree nor disagree	Agree	Strongly agree
I am afraid that incorrect statements will be associated with me personally.	☐	☐	☐	☐	☐
I find being addressed by name uncomfortable for cultural reasons.	☐	☐	☐	☐	☐
I feel more comfortable during the session.	☐	☐	☐	☐	☐
I find it uncomfortable when my name is mentioned in the seminar group.	☐	☐	☐	☐	☐
I feel better prepared for examinations.	☐	☐	☐	☐	☐
I find being addressed by name uncomfortable for personal reasons.	☐	☐	☐	☐	☐
I feel more appreciated.	☐	☐	☐	☐	☐
I have to overcome myself more to contribute.	☐	☐	☐	☐	☐
I feel a stronger sense of belonging to the learning group / course.	☐	☐	☐	☐	☐
If I say something incorrect, my fellow students think poorly of me.	☐	☐	☐	☐	☐
I can remember the content for a longer period of time.	☐	☐	☐	☐	☐

JUSTUS-LIEBIG-UNIVERSITÄT GIESSEN

	Strongly disagree	Disagree	Neither agree nor disagree	Agree	Strongly agree
I feel taken more seriously as a person.	☐	☐	☐	☐	☐
I find being addressed by name uncomfortable because I feel insecure in social situations.	☐	☐	☐	☐	☐
The learning environment feels safer.	☐	☐	☐	☐	☐
I am more focused.	☐	☐	☐	☐	☐
I have the feeling that my contributions are taken more seriously.	☐	☐	☐	☐	☐
Being addressed by name interferes with my privacy.	☐	☐	☐	☐	☐
I feel pressured to express myself.	☐	☐	☐	☐	☐
I become more interested in the subject content.	☐	☐	☐	☐	☐
I understand the content better.	☐	☐	☐	☐	☐
I do not want to disappoint the instructor with my performance.	☐	☐	☐	☐	☐
I perceive being addressed by name as intrusive.	☐	☐	☐	☐	☐
It is easier to make mistakes and ask questions.	☐	☐	☐	☐	☐
If I say something incorrect, the instructor thinks poorly of me.	☐	☐	☐	☐	☐
I feel perceived as an individual person.	☐	☐	☐	☐	☐
My confidence for future examinations increases.	☐	☐	☐	☐	☐
I prefer to remain anonymous so that I can follow the session silently.	☐	☐	☐	☐	☐

	Strongly disagree	Disagree	Neither agree nor disagree	Agree	Strongly agree
I have more conversations with fellow students outside the session due to name mention.	☐	☐	☐	☐	☐
I can reflect more on my learning progress.	☐	☐	☐	☐	☐
I become nervous that I might be called on more often.	☐	☐	☐	☐	☐
I become more aware of my own presence in the room.	☐	☐	☐	☐	☐
I have the feeling that I am evaluated more strongly by fellow students.	☐	☐	☐	☐	☐
I find it easier to ask questions.	☐	☐	☐	☐	☐
I try to comply more carefully with social norms (e.g., punctuality).	☐	☐	☐	☐	☐
I participate more frequently.	☐	☐	☐	☐	☐
I listen more attentively.	☐	☐	☐	☐	☐
I invest more time in preparation and follow-up.	☐	☐	☐	☐	☐
I come into conversation with fellow students during the session more often due to name use.	☐	☐	☐	☐	☐
I miss fewer sessions.	☐	☐	☐	☐	☐
I refrain more from distracting activities (e.g., mobile phone use).	☐	☐	☐	☐	☐
I have the feeling that I am evaluated more strongly by fellow students	☐	☐	☐	☐	☐
The threshold for addressing personal problems to the instructor decreases as a result.	☐	☐	☐	☐	☐
The session becomes more interactive as a result.	☐	☐	☐	☐	☐

JUSTUS-LIEBIG-UNIVERSITÄT GIESSEN

	Strongly disagree	Disagree	Neither agree nor disagree	Agree	Strongly agree
The instructor addresses me more often if they believe that I have knowledge gaps.	☐	☐	☐	☐	☐
I am more likely to make use of office hour offers.	☐	☐	☐	☐	☐
Being addressed by name raises my hope that advantages for me may arise during my studies (e.g., doctoral thesis, internship).	☐	☐	☐	☐	☐
My prior knowledge is taken into account more strongly.	☐	☐	☐	☐	☐
The session becomes more structured as a result.	☐	☐	☐	☐	☐
My learning achievements are praised more often.	☐	☐	☐	☐	☐
The instructor addresses me more specifically to assess my knowledge.	☐	☐	☐	☐	☐
I have the feeling that students who are known by name are preferred by instructors.	☐	☐	☐	☐	☐
The instructor appears more competent.	☐	☐	☐	☐	☐
Personal address increases my motivation to engage with the subject content.	☐	☐	☐	☐	☐
I am more likely to seek individual conversation with the instructor directly before or after the session.	☐	☐	☐	☐	☐
The instructor becomes more likeable as a result.	☐	☐	☐	☐	☐
I have the feeling that my learning success is more important to the instructor.	☐	☐	☐	☐	☐
Addressing is selective, so that my individual level of knowledge is taken into account.	☐	☐	☐	☐	☐
I am addressed more frequently.	☐	☐	☐	☐	☐
I have the feeling that the instructor wants to single me out.	☐	☐	☐	☐	☐
